# Association between secondhand smoke exposure and hyperuricemia among never smokers: A cross-sectional analysis of NHANES data

**DOI:** 10.18332/tid/220846

**Published:** 2026-07-17

**Authors:** Yahui Liang, Tian Zhou, Aoran Yang, Dan Wang, Wenyue Zhang, Weijun Gong

**Affiliations:** 1 Department of Traditional Chinese Medicine, Beijing Rehabilitation Hospital of Capital Medical University, Beijing, China; 2 Beijing Key Laboratory of Intelligent Drug Research and Development for Mental Disorders, National Clinical Research Center for Mental Disorders, National Center for Mental Disorders, Beijing Anding Hospital, Capital Medical University, Beijing, China; 3 Rehabilitation Medical College, Beijing Rehabilitation Hospital of Capital Medical University, Beijing, China

**Keywords:** cross-sectional study, hyperuricemia, NHANES, secondhand smoke, serum cotinine

## Abstract

**INTRODUCTION:**

Secondhand smoke (SHS) exposure is recognized as an important contributor to multiple adverse health outcomes, including hyperuricemia. However, the association between SHS exposure and hyperuricemia remains inconclusive. This study aimed to examine the relationship between SHS exposure and hyperuricemia using serum cotinine concentration as an objective biomarker.

**METHODS:**

Data were obtained from the National Health and Nutrition Examination Survey (NHANES) collected between 1999 and 2020. A cross-sectional analysis was performed among 18610 adults who self-reported never smoking (never smokers). Participants were categorized into four groups based on serum cotinine concentrations: non-exposure (<0.05 ng/mL), low-exposure (0.05–0.99 ng/mL), moderate-exposure (1–10 ng/mL), and heavy-exposure (>10 ng/mL). Associations between SHS exposure and hyperuricemia were evaluated using multivariable logistic regression analysis models and smooth curve fitting.

**RESULTS:**

After adjusting for potential confounders, the adjusted odds ratios (AOR) for hyperuricemia in the low-, moderate-, and heavy-exposure groups, compared with the non-exposure group, were 1.34 (95% CI: 1.18–1.53), 1.48 (95% CI: 1.08–2.02), and 1.97 (95% CI: 1.27–3.05), respectively. A non-linear positive saturation association was observed between log2-transformed serum cotinine concentrations and the prevalence of hyperuricemia. The inflection point was identified at approximately -3.37 ng/mL, with an AOR of 1.15 (95% CI: 1.08–1.22). Subgroup analysis revealed consistent associations across all demographic and clinical stratifications.

**CONCLUSIONS:**

Among never smokers, SHS exposure was significantly associated with an increased risk of hyperuricemia. A threshold effect was identified at a serum cotinine concentration of -3.37 ng/mL. These findings provide a direction for further study on the relationship between SHS exposure and hyperuricemia.

## INTRODUCTION

Hyperuricemia is a prevalent chronic metabolic disorder that contributes significantly to individual morbidity and societal healthcare burden. As of 2016, hyperuricemia affected approximately 21% of the global population, with reported prevalence estimates ranging from 14.6% to 20% in the United States^1,2^. Hyperuricemia has been identified as an independent risk factor for several systemic conditions, including hypertension, gout, cardiovascular diseases, chronic kidney disease, and all-cause mortality^3,4^.

Although numerous investigations have examined the relationship between smoking and serum uric acid levels, the findings remain inconclusive^5^. Several cross-sectional analyses have reported lower serum uric acid concentrations among individuals who currently smoked^6,7^. In contrast, exposure to electronic cigarettes in a Korean population was associated with elevated serum uric acid levels and an increased prevalence of hyperuricemia^8^. Urinary cotinine concentration, used as an objective biomarker of tobacco exposure, was positively associated with increased serum uric acid levels and hyperuricemia among adult women in Korea^9^. In addition, a long-term community-based prospective analysis indicated that current smoking status was associated with an increased incidence of hyperuricemia or gout in women^10^.

Secondhand smoke (SHS) exposure, which comprises exhaled mainstream smoke and sidestream smoke from burning tobacco products, has been linked to a wide range of adverse health effects^11^. SHS exposure has been associated with the development of various malignancies, and it exerts adverse effects on the cardiovascular system, substantially increasing the risk of stroke and heart disease^12,13^. However, limited evidence is available regarding the association between SHS exposure and hyperuricemia.

Nicotine, a primary harmful component of tobacco smoke, is metabolized into cotinine, which can be quantified in blood, urine, and saliva. Among these, serum cotinine concentration serves as an objective and reliable biomarker for assessing exposure to tobacco smoke and associated health risks^14^. The present cross-sectional analysis used serum cotinine levels to assess SHS exposure and examined its association with hyperuricemia among adults with no history of smoking in the United States.

## METHODS

### Study design and population

**Figure 1:**
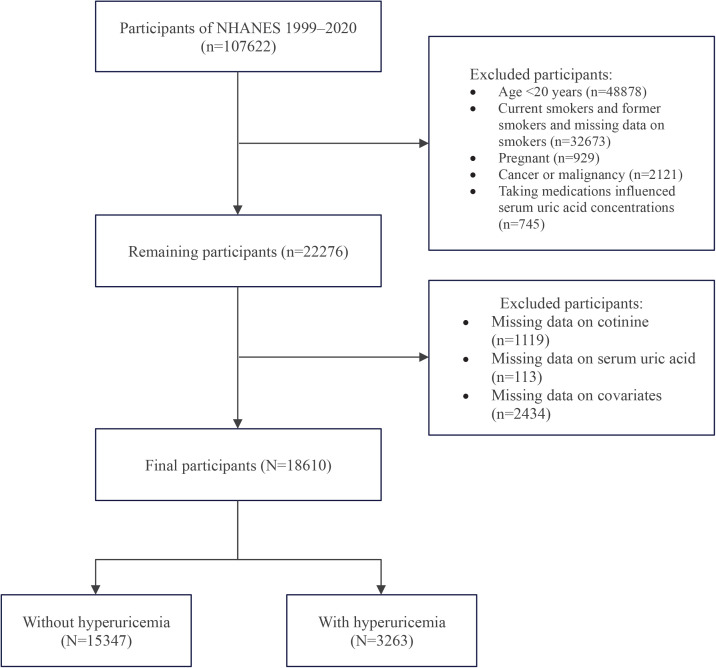
Flow diagram of participant selection from the NHANES 1999–2020 survey cycles

The National Health and Nutrition Examination Survey (NHANES), conducted by the National Center for Health Statistics (NCHS), assesses the health and nutritional status of the non-institutionalized population in the United States. The survey applies a nationally representative, stratified, multistage probability sampling design^15^. The present analysis used NHANES data collected between 1999 and 2020 (including cycles 1999–2000, 2001–2002, 2003–2004, 2005–2006, 2007–2008, 2009–2010, 2011–2012, 2013–2014, 2015–2016, and 2017–2020) and focused on participants aged ≥20 years, adults who self-reported never smoking (never smokers). Individuals were excluded if they were pregnant, had a history of malignancy, were using medications known to influence serum uric acid levels, or had used nicotine-containing products within 5 days preceding the survey. Participants with missing data on serum cotinine, serum uric acid, or relevant covariates were also excluded. The participant selection process is illustrated in Figure 1.

### Assessment of smoking status and tobacco smoke exposure

Individuals were classified as never smokers if they reported smoking fewer than 100 cigarettes during their lifetime. Consistent with previous research and evidence indicating that serum cotinine concentrations >10 ng/mL are typical among most active smokers, SHS exposure was classified into four categories^16,17^. Participants were classified as follows based on serum cotinine concentrations: non-exposure <0.05 ng/mL), low-exposure (0.05–0.99 ng/mL), moderate-exposure (1–10 ng/mL), or heavy-exposure (>10 ng/mL)^18^.

### Assessment of hyperuricemia

Hyperuricemia was determined based on serum uric acid concentrations obtained from laboratory measurements. Blood samples were collected at mobile examination centers and stored at -20°C until analysis. Serum uric acid levels were measured by trained laboratory technicians at Collaborative Laboratory Services in Ottumwa, Iowa, using the timed-endpoint method with the Beckman Colter UNICEL DxC 800 analyzer. Hyperuricemia was defined as a serum uric acid concentration of ≥416 μmol/L (7.0 mg/dL) in men and ≥357 μmol/L (6.0 mg/dL) in women^19^.

### Covariates

NHANES data were collected using standardized questionnaires administered in participants’ homes and comprehensive medical examinations. In accordance with prior literature, the covariates taken into account in this study encompassed age, gender (male, female), race/ethnicity (non-Hispanic White, non-Hispanic Black, Mexican American, Other Hispanic, Other race including multi-racial), education level (lower than high school, high school or equivalent, some college or higher), marital status (married/living with partner vs widowed/divorced/separated/never married), body mass index (BMI) (kg/m^2^), family income categorized by poverty income ratio (PIR: low ≤1.3, medium 1.3–3.5, high >3.5), physical activity, alcohol drinking status, hypertension, diabetes, coronary heart disease, stroke, chronic kidney disease, estimated glomerular filtration rate (eGFR), serum uric acid, and hyperuricemia. Physical activity was quantified by multiplying the duration of weekly activities by their corresponding metabolic equivalents (MET) values. Physical activity was categorized by total MET score, with values <600 per week indicating low activity, 600–3000 indicating moderate activity, and >3000 indicating vigorous activity^20^. Alcohol consumption was categorized as never drinkers (lifetime consumption <12 occasions), former drinkers (≥12 occasions in the past year but not currently drinking), and current drinkers (≥12 occasions in the past year). Self-reported histories of hypertension, diabetes mellitus, and other chronic diseases were determined using physician-reported information from the NHANES questionnaire.

### Statistical analysis

This secondary analysis was conducted using publicly available data from NHANES between 1999 and 2020 (including cycles 1999–2000, 2001–2002, 2003–2004, 2005–2006, 2007–2008, 2009–2010, 2011–2012, 2013–2014, 2015–2016, and 2017–2020). Analyses incorporated NHANES sampling weights to account for the complex survey design. Following NHANES official guidelines for pooled analyses, the sampling weights for 1999–2020 were calculated as follows: 1999–2002 weights were 2/10.6 WTMEC4YR; 2003–2016 weights were 1/10.6 WTMEC2YR; 2017–2020 weights were 1.6/10.6 WTMECPRP. A complex survey design object was constructed using the *survey* package (version 4.2.2) in R, specifying primary sampling units (SDMVPSU) and stratification variables (SDMVSTRA). All logistic regression models were fitted using *svyglm()* with *family=quasibinomial()* to obtain survey-weighted adjusted odds ratios (AORs) and 95% confidence intervals (CIs).

To assess multicollinearity among covariates, variance inflation factors (VIF) were calculated for all predictor variables in the final multivariable model. All VIF values were well below the conventional threshold of 5 (range: 1.05–1.39), confirming no substantial multicollinearity and ensuring the stability of effect estimates.

Categorical variables are presented as unweighted numbers (weighted percentages), whereas continuous variables are described as means (standard error) or medians with interquartile ranges, depending on data distribution. Differences among groups were evaluated using analysis of variance, the Kruskal–Wallis test, or the chi-squared test, as appropriate.

Multivariable logistic regression models were applied to estimate adjusted odds ratios (AORs) and corresponding 95% confidence intervals (CIs) for the association between SHS exposure and hyperuricemia.

Three sequential models were constructed. Model 1 adjusted for sociodemographic variables, including age, sex, race or ethnicity, marital status, education level, and family income. Model 2 adjusted as for Model 1 plus BMI, physical activity level, and alcohol consumption status. Model 3 adjusted as for Model 2 plus hypertension, coronary heart disease, diabetes, stroke, chronic kidney disease, and eGFR.

The potential nonlinear dose-response association between log2-transformed serum cotinine concentrations and hyperuricemia was evaluated using survey-weighted restricted cubic spline regression models with four knots, adjusted for the covariates included in the final multivariable model.

To identify the optimal inflection point for the non-linear relationship between serum cotinine and hyperuricemia while accounting for the NHANES complex sampling design, we employed a weighted segmented regression approach using the *survey* package in R (version 4.2.2). We systematically searched for the threshold across a range of log2-transformed cotinine concentrations (from -4.0 to -2.0, corresponding to 0.063–0.25 ng/mL in original scale) with increments of 0.05. At each candidate threshold, participants were dichotomized into low- and high-exposure segments, and a weighted quasi-binomial regression model was fitted incorporating the linear term (centered at the threshold), the segment indicator, and their interaction term (cotinine × segment), adjusting for age, sex, BMI, race/ethnicity, education level, family income, physical activity, alcohol consumption, hypertension, diabetes, coronary heart disease, stroke, chronic kidney disease, and eGFR. The optimal threshold was determined as the value that minimized the p-value of the interaction term, indicating the most significant difference in slopes between the two segments. To ensure statistical stability, we required a minimum of 100 observations in each segment for any candidate threshold to be considered valid. The final weighted segmented regression model was then fitted using this optimal threshold to estimate odds ratios and 95% confidence intervals for the association between the threshold and outcomes. Likelihood ratio tests were used to compare the segmented model against a simple linear model.

Subgroup analyses were conducted using survey-weighted stratified multivariable logistic regression to assess potential effect modification by age, sex, BMI category, hypertension, and diabetes status, with interaction terms evaluated using likelihood ratio tests.

All statistical analyses were performed using R Statistical Software (Version 4.2.2; The R Foundation, Vienna, Austria) and the Free Statistics analysis platform (Version 2.1; Beijing, China). Statistical significance was defined as a two-sided p<0.05.

## RESULTS

### Study population

Data from NHANES cycles between 1999 and 2020 were analyzed for individuals aged ≥20 years who self-reported as never smokers (n=26071). Individuals who were pregnant, had cancer or other malignancies, or were using hypouricemic agents (allopurinol or febuxostat) or diuretics (hydrochlorothiazide or furosemide) known to affect serum uric acid concentrations were excluded (n=3795)^21^. Participants with missing data on serum cotinine (n=1119), serum uric acid (n=113), or other covariates (n=2434) were also excluded. The final analytic sample comprised 18610 participants. Details of the participant selection process are presented in Figure 1.

### Baseline characteristics

Baseline characteristics of the participants stratified by serum cotinine concentration are presented in Table 1. Hyperuricemia was identified in 3263 participants (17.5%). The mean age of the study population was 44.8 years (SE=0.24), and 42.24% of participants (n=7544) were male. Compared with individuals in the SHS exposure groups, those in the non-exposure group were more likely to be female (p<0.001), married or living with a partner (p<0.001), possess higher level of education (p<0.001), report higher family income (p<0.001), and demonstrate a lower prevalence of hyperuricemia (p<0.001).

**Table 1:** Baseline characteristics of the participants in the NHANES 1999–2020 cycles

	*SHS exposure (serum cotinine levels, ng/mL)*					
*Characteristics*	*Total n (%)*	*Non-exposure (<0.05) n (%)*	*Low exposure (0.05–0.99) n (%)*	*Moderate exposure (1–10) n (%)*	*Heavy exposure (>10) n (%)*	*p*
**Total,** n	18610	12770	4903	646	291	
**Age** (years), mean (SE)	44.48 (0.24)	45.83 (0.29)	41.61 (0.32)	38.38 (0.79)	37.85 (1.25)	<0.001
**Gender**						<0.001
Male	7544 (42.24)	4915 (40.48)	2171 (46.45)	306 (45.96)	152 (53.04)	
Female	11066 (57.76)	7855 (59.52)	2732 (53.55)	340 (54.04)	139 (46.96)	
**Race/ethnicity**						<0.001
Non-Hispanic White	7046 (64.75)	5052 (67.51)	1719 (59.01)	221 (56.11)	54 (38.14)	
Non-Hispanic Black	3955 (11.26)	1996 (7.79)	1508 (18.03)	288 (28.08)	163 (38.41)	
Mexican American	3681 (9.72)	2830 (10.44)	771 (8.36)	54 (4.93)	26 (6.18)	
Other	3928 (14.28)	2892 (14.26)	905 (14.60)	83 (10.88)	48 (17.28)	
**Education level** (years)						<0.001
<9	1981 (4.82)	1398 (4.54)	502 (5.62)	56 (5.17)	25 (4.31)	
9–12	5753 (27.46)	3379 (22.95)	1907 (37.00)	309 (47.14)	158 (53.16)	
>12	10876 (67.72)	7993 (72.51)	2494 (57.38)	281 (47.69)	108 (42.53)	
**Marital status**						<0.001
Married/living with a partner	11530 (65.88)	8450 (69.77)	2656 (57.55)	296 (48.38)	128 (46.88)	
Living alone	7080 (34.12)	4320 (30.23)	2247 (42.45)	350 (51.62)	163 (53.12)	
**Family income**						<0.001
Low	4971 (17.77)	2914 (14.17)	1637 (24.92)	286 (37.05)	134 (38.72)	
Medium	6887 (33.39)	4659 (32.05)	1871 (36.38)	248 (40.37)	109 (34.79)	
High	6752 (48.84)	5197 (53.77)	1395 (38.70)	112 (22.58)	48 (26.49)	
**BMI** (kg/m^2^), mean (SE)	28.83 (0.08)	28.51 (0.09)	29.62 (0.15)	30.27 (0.32)	27.75 (0.46)	<0.001
**Physical activity**						<0.001
Low	9040 (44.95)	5996 (42.70)	2580 (50.85)	326 (50.74)	138 (44.98)	
Moderate	5411 (32.05)	3948 (34.10)	1256 (27.18)	142 (23.76)	65 (29.22)	
Vigorous	4159 (23.00)	2826 (23.20)	1067 (21.96)	178 (25.50)	88 (25.80)	
**Alcohol drinking status**						0.22
Never	6736 (29.90)	4718 (29.87)	1763 (30.71)	170 (26.45)	85 (24.09)	
Former/current	11874 (70.10)	8052 (70.13)	3140 (69.29)	476 (73.55)	206 (75.91)	
**Health status**						
Hypertension	4415 (20.46)	3051 (20.51)	1159 (20.78)	148 (19.07)	57 (14.47)	0.27
Diabetes	1845 (6.97)	1312 (6.99)	446 (6.80)	53 (6.53)	34 (10.11)	0.54
Coronary heart disease	396 (1.68)	291 (1.74)	90 (1.64)	12 (1.17)	3 (0.34)	0.21
Stroke	390 (1.60)	274 (1.64)	93 (1.43)	15 (2.29)	8 (1.25)	0.38
Chronic kidney disease	359 (1.55)	234 (1.48)	99 (1.53)	13 (1.92)	13 (5.44)	0.003
eGFR (CKD-EPI), mean (SE)	98.43 (0.30)	97.59 (0.35)	100.38 (0.39)	101.96 (1.09)	100.46 (1.79)	<0.001
Serum uric acid (mg/dL), mean (SE)	5.27 (1.37)	5.20 (1.34)	5.47 (1.43)	5.44 (1.49)	5.39 (1.52)	<0.001
Hyperuricemia	3263 (17.03)	2039 (15.45)	1013 (20.72)	148 (22.40)	63 (23.05)	<0.001

BMI: body mass index. SHS: secondhand smoke. CKD-EPI: Chronic Kidney Disease Epidemiology Collaboration. Categorical variables are summarized as unweighted numbers (weighted percentage), and continuous variables are expressed as mean (standard error). Differences among groups were evaluated using analysis of variance, the Kruskal–Wallis test, or the chi-squared test, as appropriate. A p<0.05 was considered statistically significant (two-tailed test).

### Association between SHS exposure and hyperuricemia

Multivariable logistic regression results are summarized in Table 2. After adjustment for all potential confounders, SHS exposure was positively associated with hyperuricemia. Compared with the non-exposure group (serum cotinine concentration <0.05 ng/mL), the adjusted odds ratios (AORs) for hyperuricemia were: 1.34 (95% CI: 1.18–1.53, p<0.001) in the low-exposure group (0.05–0.99 ng/mL), 1.48 (95% CI: 1.08–2.02, p=0.014) in the moderate-exposure group (1–10 ng/mL), and 1.97 (95% CI: 1.27–3.05, p=0.03) in the heavy-exposure group (>10 ng/mL).

**Table 2:** Multivariate logistic regression analysis of the relationship between SHS exposure and hyperuricemia in never smoking adults among the participants in the NHANES 1999–2020 cycles

*Variables*	*Total n*	*Crude Model OR (95% CI)*	*p*	*Model 1 AOR (95% CI)*	*p*	*Model 2 AOR (95% CI)*		*Model 3 AOR (95% CI)*	*p*
**Log2-transformed cotinine** (ng/mL)	18610	1.07 (1.05–1.10)	<0.001	1.07 (1.05–1.10)	<0.001	1.07 (1.05–1.10)	<0.001	1.07 (1.05–1.10)	<0.001
**SHS exposure**									
Non-exposure (ref.)	12770	1		1		1		1	
Low exposure	4903	1.43 (1.28–1.60)	<0.001	1.42 (1.26–1.60)	<0.001	1.34 (1.19–1.52)	<0.001	1.34 (1.18–1.53)	<0.001
Moderate exposure	646	1.58 (1.21–2.07)	0.001	1.59 (1.22–2.08)	0.001	1.48 (1.19–1.8)	0.012	1.48 (1.08–2.02)	0.014
Heavy exposure	291	1.64 (1.08–2.49)	0.021	1.61 (1.03–2.52)	0.021	2.02 (1.27–3.20)	0.003	1.97 (1.27–3.05)	0.003
Trend.test			<0.001		<0.001		<0.001		<0.001

SHS: secondhand smoke. AOR: adjusted odds ratio. Model 1 was adjusted for demographic and socioeconomic factors, including age, gender, race/ethnicity, marital status, education level, and family income. Model 2 was adjusted as for Model 1 plus BMI, physical activity, and alcohol consumption status. Model 3 was adjusted as for Model 2 plus hypertension, diabetes, coronary heart disease, stroke, chronic kidney disease, and eGFR.

**Figure 2:**
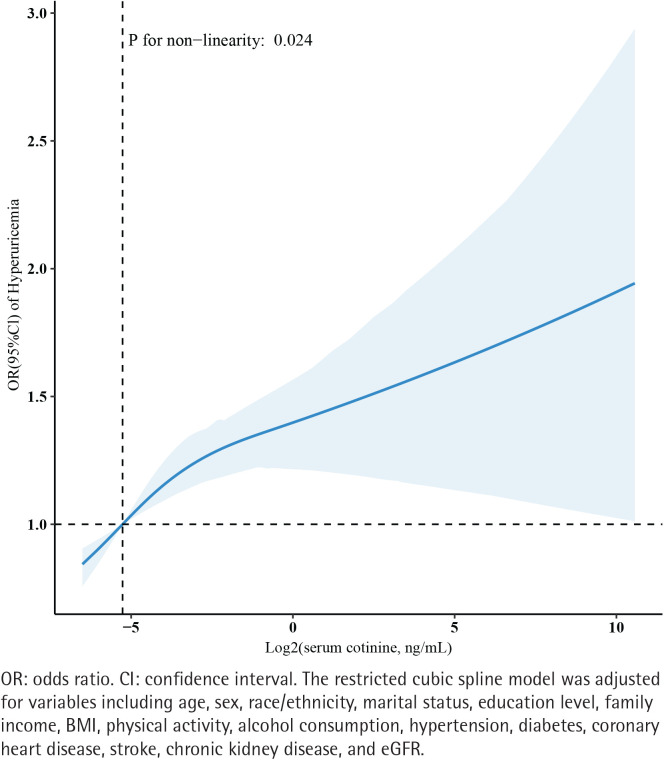
Non-linear association between log2-transformed serum cotinine concentrations and hyperuricemia among never smokers

Restricted cubic spline analysis demonstrated a non-linear, positive saturation association between log2-transformed serum cotinine concentrations and the odds of hyperuricemia (p for non-linearity=0.024) (Figure 2).

Threshold analysis identified an inflection point at a log2-transformed serum cotinine concentration of -3.37 ng/mL. Below this threshold, each one-unit increase in log2-transformed serum cotinine concentration was associated with a significantly elevated odds of hyperuricemia (AOR=1.15; 95% CI: 1.08–1.22, p<0.001). Above this threshold, no statistically significant association with hyperuricemia was observed (AOR=1.04; 95% CI: 0.99–1.08, p=0.135) (Table 3).

**Table 3:** Segmented regression analysis of the relationship between serum cotinine levels and hyperuricemia of the participants in the NHANES 1999–2020 cycles

*Log2-transformed cotinine (ng/mL)*	*AOR (95% CI)*	*p*
< -3.37	1.15 (1.08–1.22)	<0.001
≥ -3.37	1.04 (0.99–1.08)	0.135
**Likelihood Ratio test**		0.001

AOR: adjusted odds ratio. The analysis controlled for variables age, gender, race/ethnicity, marital status, education level, family income, BMI, physical activity, alcohol consumption, hypertension, diabetes, coronary heart disease, stroke, chronic kidney disease, and eGFR.

### Subgroup analyses

**Figure 3:**
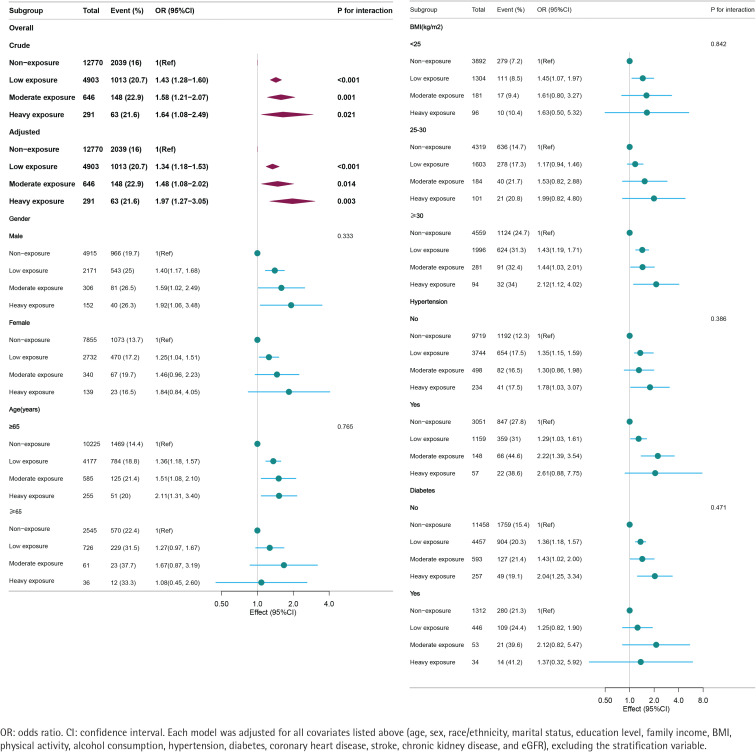
Stratified associations between secondhand smoke exposure and hyperuricemia among never smokers

Stratified analyses were conducted to evaluate potential effect modification in the association between SHS exposure and hyperuricemia across subgroups defined by age, sex, BMI, hypertension, and diabetes status. No statistically significant interactions were observed across these strata (Figure 3).

## DISCUSSION

This cross-sectional analysis of adult never smokers in the United States identified a non-linear, positive saturation association between SHS exposure and hyperuricemia. When log2-transformed serum cotinine concentrations were below -3.37 ng/mL, each one-unit increase was associated with a 15% increase in the risk of hyperuricemia. No statistically significant association was observed at cotinine concentrations exceeding -3.37 ng/mL. These findings indicate that SHS exposure, even at lower levels, is associated with an increased risk of hyperuricemia, and that the strength of this association may plateau beyond a certain exposure level. Subgroup analyses further indicated that this association remained stable across examined strata.

Smoking represents a major preventable cause of mortality worldwide, contributing to approximately 8 million deaths annually, including an estimated 1.3 million deaths attributable to SHS exposure^22^. SHS has been known to contain higher concentrations of several harmful substances compared with mainstream smoke directly inhaled by active smokers. Inhalation of these substances has been associated with a wide range of adverse health outcomes, including respiratory disorders, metabolic abnormalities, cardiovascular diseases, and multiple malignancies^23^.

Previous investigations examining the association between smoking and serum uric acid levels have produced inconsistent findings. Several methodological factors may contribute to these discrepancies. First, differences in study population characteristics are substantial. Previous cross-sectional studies reporting inverse associations often included former smokers in their ‘non-smoking’ reference groups or analyzed mixed populations of current, former, and never smokers^24,25^, whereas our study exclusively focused on never smokers. This distinction is critical because former smokers may exhibit different metabolic profiles compared with never smokers even after smoking cessation^26^. Second, sample size variations significantly impact statistical power. Several studies demonstrating null or inverse associations were limited by relatively small sample sizes (n<5000), which may have been underpowered to detect modest associations^6,7^. Our analysis of 18610 never smokers provides robust statistical power to detect significant associations. Third, exposure assessment methodologies differ considerably. Previous studies primarily relied on self-reported smoking status, which is susceptible to recall bias and social desirability bias, potentially leading to exposure misclassification^17^. The use of serum cotinine as an objective biomarker in our study minimizes such misclassification and allows for quantification of SHS exposure levels. Fourth, variations in covariate adjustment strategies may explain discrepant findings. While some studies adjusted for limited confounders (e.g. age and sex only)^6^, our multivariable models comprehensively adjusted for demographic, socio-economic, lifestyle, and clinical factors, including eGFR, which is critical given renal mechanisms underlying uric acid metabolism^27^.

Several studies have reported associations between smoke exposure and reduced serum uric acid concentrations. For example, Haj et al.^24^ reported lower serum uric acid levels among current smokers compared with non-smokers, with a stronger inverse association observed in men. However, their definition of non-smokers includes former smokers, which differs from the never smoker population analyzed in the present study^24^. Teng et al.^25^ found that smoking was associated with a lower incidence of gout in men, and that serum uric acid levels were lower in current smokers compared to former smokers and never smokers, with significant associations observed only in men. Yang et al.^7^ further reported a negative association between smoking and hyperuricemia among middle-aged and older men, with no significant association observed among women^7^. Additionally, increased oxidative stress resulting from SHS exposure may lead to enhanced consumption of uric acid, which functions as a major antioxidant^28^. Consistent with this explanation, passive smokers were reported to exhibit lower serum antioxidant levels than those unexposed to SHS^28^.

In contrast to studies reporting inverse associations, several studies have reported positive associations between tobacco smoke exposure and elevated serum uric acid levels. A community-based prospective study conducted by Teramura et al.^10^ indicated that smoking status was significantly associated with the development of hyperuricemia among women, while in men, hypertension and alcohol intake were more strongly associated with hyperuricemia. Yang et al.^7^ reported that male users of both combustible and electronic cigarettes exhibited higher serum uric acid levels than non-smokers, and that women who were single-product users also had higher serum uric acid levels than their non-smoking counterparts. Kim et al.^8^ reported that male users of electronic cigarettes in South Korea exhibited higher serum uric acid and high-sensitivity C-reactive protein levels compared to non-smokers, with uric acid levels exceeding those observed in individuals who exclusively smoked traditional cigarettes^8^. Kim et al.^8^ reported that exposure to electronic cigarettes was associated with increased serum uric acid levels and hyperuricemia, with dual use of electronic and traditional cigarettes potentially exerting a more pronounced adverse effect on uric acid metabolism.

SHS, defined as the involuntary inhalation of tobacco smoke exhaled by users or released from burning tobacco products, contains higher concentrations of toxic and carcinogenic compounds in sidestream smoke than in mainstream smoke inhaled directly^29^. Among never smokers, SHS exposure represents a significant source of environmental tobacco exposure and has been increasingly recognized as a contributor to adverse health outcomes, including hyperuricemia. However, evidence regarding the association between SHS exposure and hyperuricemia remains limited. Han et al.^6^ reported that individuals with hyperuricemia were more frequently exposed to SHS than those with normal serum uric acid levels, and that weekly exposure for more than one year was associated with a 2.6-fold increased risk of hyperuricemia^6^. Additional studies also indicated that passive smoking was associated with an increased risk of hyperuricemia^30^.

Several biological mechanisms may explain the observed association between SHS exposure and elevated serum uric acid levels. SHS contains nicotine, polycyclic aromatic hydrocarbons, carbon monoxide, and heavy metals, which may influence uric acid metabolism through multiple pathways. First, these substances increase oxidative stress, which can disrupt uric acid metabolism and excretion. Oxidative damage may promote intracellular purine release, resulting in increased uric acid production^31^. Second, SHS exposure may contribute to systemic inflammation. Nicotine has pro-inflammatory effects, including the promotion of endothelial dysfunction. A randomized crossover trial reported increased levels of myeloperoxidase, an inflammatory marker, following exposure to nicotine-containing electronic cigarettes^32^. Evidence indicates that systemic inflammation mediates the association between SHS exposure and increased serum uric acid levels^33^. Inflammation-induced activation of the renin-angiotensin system may further impair renal uric acid excretion^31^. Third, exposure to heavy metals such as lead and cadmium present in SHS has been associated with increased serum uric acid levels and an elevated risk of hyperuricemia in epidemiological studies^34^. These proposed mechanisms warrant further investigation.

### Strengths and limitations

This analysis incorporated multiple NHANES survey cycles, allowing for a large and nationally representative sample with substantial racial and ethnic diversity, which enhances the generalizability of the findings to the US adult population. Assessment of never smoker status using both self-reported information and serum cotinine concentrations reduced misclassification and strengthened the validity of exposure assessment.

Nevertheless, several limitations should be acknowledged. First, residual confounding may exist due to unmeasured variables, including dietary factors^30^ (e.g. purine and fructose intake), genetic polymorphisms affecting uric acid metabolism (e.g. SLC2A9, ABCG2), and occupational exposures. Second, recall and social desirability bias may affect self-reported data on alcohol consumption, physical activity, and disease history^35^; although serum cotinine verification strengthened smoking classification, other variables remain susceptible to reporting bias. Third, missing data may introduce selection bias^36^. We excluded 7461 participants (28.6%) due to incomplete data, and if these were not missing completely at random (MCAR), results could be biased (compared with included participants, excluded individuals were older and more likely non-Hispanic Black). Fourth, the heavy exposure group (>10 ng/mL) comprised only 291 participants (1.6%), yielding wide confidence intervals and limiting precision for high-dose estimates. Fifth, generalizability is limited; the findings apply to US never smokers aged ≥20 years (excluding pregnant women and cancer patients) and may not extend to other populations; the 1999–2020 timeframe may also introduce period effects from changing smoking policies. Finally, the cross-sectional design precludes causal inference; serum cotinine’s short half-life (16–20 hours) reflects recent rather than cumulative exposure, and thirdhand smoke may contribute to cotinine levels^16,17^.

## CONCLUSIONS

This cross-sectional analysis of never smokers in the United States identified a significant positive association between SHS exposure and hyperuricemia. A non-linear dose–response relationship was observed, with a distinct threshold at a log2-transformed serum cotinine concentration of approximately -3.394 ng/mL. These findings indicate that even low levels of SHS exposure may be associated with an increased risk of hyperuricemia among never smokers. Further prospective, longitudinal studies are required to confirm these associations and to clarify the biological mechanisms underlying the observed relationships.

## Data Availability

The data supporting this research are available from the following sources NHANES website (https://www.cdc.gov/nchs/nhanes/).
